# Research on Ocular Artifacts Removal from Single-Channel Electroencephalogram Signals in Obstructive Sleep Apnea Patients Based on Support Vector Machine, Improved Variational Mode Decomposition, and Second-Order Blind Identification

**DOI:** 10.3390/s24051642

**Published:** 2024-03-02

**Authors:** Xin Xiong, Zhiran Sun, Aikun Wang, Jiancong Zhang, Jing Zhang, Chunwu Wang, Jianfeng He

**Affiliations:** 1 Faculty of Information Engineering and Automation, Kunming University of Science and Technology, Kunming 650500, China; xiongxin@kust.edu.cn (X.X.); 20212204289@stu.kust.edu.cn (Z.S.); 20212204005@stu.kust.edu.cn (A.W.); 20212104115@stu.kust.edu.cn (J.Z.); 20212204149@stu.kust.edu.cn (J.Z.); 2 College of Physics and Electronic Engineering, Hanshan Normal University, Chaozhou 521041, China; chunwu@163.com

**Keywords:** electroencephalogram, ocular artifact, GAVMD, SOBI, sleep staging

## Abstract

The electroencephalogram (EEG) has recently emerged as a pivotal tool in brain imaging analysis, playing a crucial role in accurately interpreting brain functions and states. To address the problem that the presence of ocular artifacts in the EEG signals of patients with obstructive sleep apnea syndrome (OSAS) severely affects the accuracy of sleep staging recognition, we propose a method that integrates a support vector machine (SVM) with genetic algorithm (GA)-optimized variational mode decomposition (VMD) and second-order blind identification (SOBI) for the removal of ocular artifacts from single-channel EEG signals. The SVM is utilized to identify artifact-contaminated segments within preprocessed single-channel EEG signals. Subsequently, these signals are decomposed into variational modal components across different frequency bands using the GA-optimized VMD algorithm. These components undergo further decomposition via the SOBI algorithm, followed by the computation of their approximate entropy. An approximate entropy threshold is set to identify and remove components laden with ocular artifacts. Finally, the signal is reconstructed using the inverse SOBI and VMD algorithms. To validate the efficacy of our proposed method, we conducted experiments utilizing both simulated data and real OSAS sleep EEG data. The experimental results demonstrate that our algorithm not only effectively mitigates the presence of ocular artifacts but also minimizes EEG signal distortion, thereby enhancing the precision of sleep staging recognition based on the EEG signals of OSAS patients.

## 1. Introduction

The electroencephalogram (EEG), a predominant tool in analyzing brain activity and diagnosing various diseases, facilitates recording the brain’s spontaneous potential activity, thus illustrating a correlation between brain activity and behavioral cognition. EEG data acquisition is typically categorized into invasive methods and non-invasive methods [1]. In medical practice, more accurate EEG signals are obtained by implanting electrodes into specific brain regions using invasive methods. Although this method allows for the collection of cleaner and more effective EEG signals, it involves certain risks and is complex [2,3]. Non-invasive EEG signal acquisition methods have partially mitigated this issue; however, these approaches are prone to interference from physiological artifacts during signal acquisition. The most common artifacts arise from electrocardiography (ECG), electromyography (EMG), and electrooculography (EOG) [4]. Among these, the ocular artifact has the most significant influence on the EEG signal. The ocular artifact has the characteristics of high amplitude, wide overlap with the EEG spectrum, and strong randomness, which significantly increases the difficulty of EEG signal preprocessing [5,6]. Hence, it is crucial to precisely detect and eliminate ocular artifacts before EEG data analysis [7].

In recent years, researchers have developed numerous ocular artifact removal algorithms for multi-channel EEG signals, achieving significant improvements. However, as the technology continues to evolve, the use of EEG signal acquisition and processing technology in healthcare is increasing. To lower computational complexity and enhance system portability, numerous EEG systems have shifted to using a single EEG channel, such as sleep monitoring and portable anesthesia depth monitoring [8,9]. Consequently, researching few-channel, particularly single-channel, automatic ocular artifact removal algorithms holds significant importance for the application of portable acquisition systems.

Ocular artifact processing methods are primarily categorized into data discarding and artifact elimination methods [10]. The simplest method, data discarding, involves manually inspecting acquired EEG signals and discarding segments contaminated with ocular artifacts. However, this approach may lead to significant loss of EEG information, potentially introducing more critical issues than those caused by using EEG signals with ocular artifacts [11]. Artifact elimination methods, which identify and remove artifacts while preserving the original signal’s neural information, fall into two main categories [12]. The first category involves filtering and regression; since ocular artifacts predominantly exist in the low-frequency band, they can be addressed with high-pass filtering [13]; regression-based methods necessitate reference signals that contain features of artifact signals, typically eliminating artifact interferences mixed within EEG signals through the relationship between these reference signals and the EEG signals. However, the effectiveness of this method is contingent on the quality of the reference channel’s data [14] and the potential for bidirectional contamination between the EEG and reference signals [15]. Linear filtering methods remove noise by directly limiting the spectral components associated with artifacts in the frequency domain [16]. However, due to the spectral overlap between artifacts and EEG signals, removing artifact components may also eliminate important signal components [17]. The second category involves separating or decomposing EEG and artifact signals into other domains. Among these methods, the most widely used is the blind source separation (BSS) [18] algorithm. Its fundamental principle assumes that signals from all electrodes are produced by specific sources. The observed signal can generate multiple sources after undergoing linear transformation, and the elimination of ocular artifacts is achieved by removing the sources associated with the ocular artifacts. However, given the BSS algorithm’s prerequisite that the number of channels must surpass the number of sources, this method cannot be directly applied to single-channel EEG signal processing [19]. Consequently, researchers introduced a signal decomposition approach wherein the original EEG signal is broken down into multiple components for input into the BSS, followed by identifying and removing artifact signals.

Mijovic et al. [20] combined EMD with ICA; firstly, EMD was used to decompose the EEG signal. The ICA algorithm was used to process the decomposed signal, which effectively removed artifact signals in single-channel EEG signals, but modal aliasing occurs in signal decomposition using EMD, which can easily lead to incomplete artifact removal or mistakenly removing the useful information. Subsequently, its improved algorithm, EEMD [21], was proposed; when using EEMD combined with BSS for the removal of artifacts in single-channel EEG, there is still the phenomenon of noise residuals. ZY Liu et al. [22] proposed a single-channel EEG signal ocular artifact removal algorithm based on wavelet transform, EEMD, and ICA, which effectively removes ocular artifacts and solves the problem of the artifact removal process of the WT-ICA algorithm. Still, the removal effect depends on the selection of wavelet bases and decomposition levels, which can only rely on the empirical selection of wavelet bases. In recent years, the singular spectrum analysis (SSA) technique has also been successfully used to remove artifacts from single-channel EEG signals [23]; although it can remove artifacts from EEG signals, this method can only be performed under the assumption that the EEG is smooth, and it is not applicable to longer EEG cycles.

Furthermore, the rapid advancement of machine learning technology has fostered its application in EEG artifact detection and removal, with automated recognition, multi-task learning, and flexibility showing great potential for its use. As a supervised learning model extensively utilized in pattern recognition and classification, SVM exhibits strong generalization capabilities, effectively handles high-dimensional data and feature selection issues, and its robustness confers notable advantages in EEG artifact signal recognition. Ajay et al. [24] employed an SVM to classify EEG components following source decomposition, successfully identifying artifact components and achieving high recognition accuracy. Han et al. [25] achieved artifact recognition in prefrontal EEG signals using an SVM, attaining an accuracy exceeding 95%, confirming the SVM’s effectiveness in artifact recognition applications. Yin et al. [26] introduced a deep network denoising technique based on frequency information enhancement that effectively eliminated ocular artifact components. However, this deep learning approach necessitates extensive datasets and entails lengthy training periods.

Given that ocular artifacts predominantly exist in low-frequency bands, we propose a method integrating an SVM with GA-optimized VMD and SOBI to address the limitations of existing approaches. The method proposed herein employs a dual-decomposition and dual-recognition strategy, effectively resolving the issue of incomplete separation between EEG and ocular components and minimizing information loss in regions not contaminated by artifacts.

Initially, the SVM is employed to identify artifact-contaminated segments within preprocessed EEG signals. The artifact-contaminated segments identified are subsequently decomposed into multiple Variational Mode Functions (VMFs) using a genetic algorithm (GA)-optimized variational mode decomposition (GA-VMD) algorithm. Introduced by Dragomiretskiy et al. [27] in 2014, VMD possesses adaptive characteristics, avoiding the limitations found in EMD and presenting superior noise robustness [28,29]. A previous study [30] demonstrated VMD’s efficacy in removing baseline drifts in pulse wave signals, effectively minimizing distortion. Furthermore, another research study [31] amalgamated variational mode decomposition with wavelet thresholding as an effective approach to denoise ECG signal myoelectric interference, showcasing promising denoising results. Considering the advantages of the VMD method in handling complex nonlinear, non-stationary, and multiscale signals, this study applies it to EEG signal denoising. Nevertheless, given its numerous parameters, this study further integrates a genetic algorithm for optimization.

Subsequently, the decomposed data were further processed using the SOBI algorithm. Compared to ICA and most BSS methods, SOBI is considered a superior approach [12,32]. SOBI is a method based on second-order statistics (SOS), utilizing the joint approximate diagonalization of covariance matrices to achieve blind source separation of observed signals. The approximate entropy of each component is calculated. Components with ocular artifacts are identified and removed based on the approximate entropy threshold. Ultimately, the “clean” EEG signal is reconstructed through inverse SOBI and VMD.

To demonstrate the effectiveness of the proposed method in removing ocular artifacts from single-channel EEG, experimental evaluations were conducted on both simulated and real EEG data. Furthermore, the performance of this study’s proposed method was benchmarked against four established methods.

The structure of this paper is organized as follows: Section 2 outlines the methodology employed in this study. Section 3 elaborates on the experimental data. Section 4 showcases the experimental results for simulated and real data, followed by a detailed analysis and discussion. Finally, the work is summarized.

## 2. Materials and Methods

**Figure 1:**
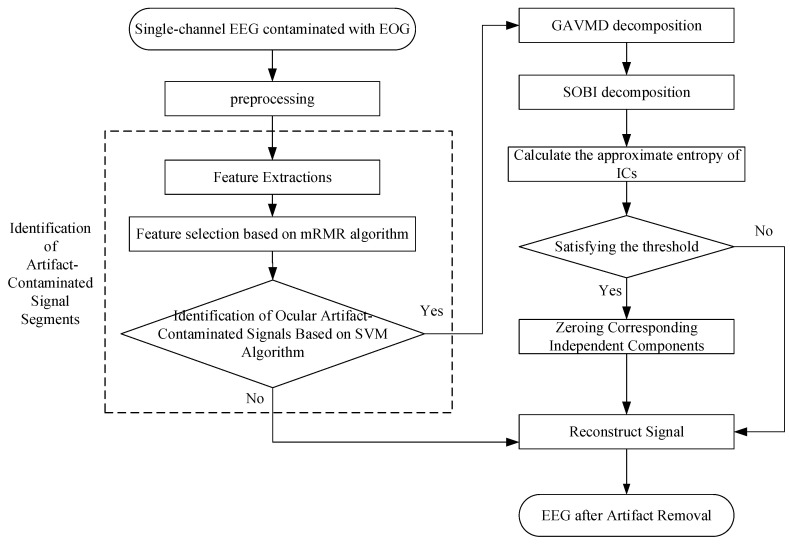
Algorithm flowchart.

Aimed at the problem of ocular artifact removal from single-channel EEG signals, we propose a method integrating an SVM with GA-optimized VMD and SOBI. Initially, the original data undergo preprocessing, and the SVM is used to identify signals contaminated with artifacts accurately; subsequently, these identified signals are dissected into more manageable fragments through the refined VMD algorithm, and the SOBI algorithm and an approximate entropy threshold is used to remove of components correlated with ocular artifacts; finally, inverse SOBI and inverse VMD algorithms are utilized to reconstruct “clean” EEG signals. Figure 1 shows a flow chart of the removal of ocular artifacts from single-channel EEG signals.

### 2.1. Identification of Contaminated Signals with Ocular Artifacts

A critical step in removing ocular artifacts is precisely identifying contaminated segments. In this study, we employ a pre-trained SVM-based classifier to detect and categorize segments tainted with ocular artifacts. The methodology unfolds as follows: Initially, the EEG signal is segmented into 10 s segments, and the time domain (including attributes such as skewness, kurtosis, and the Hjorth parameter [33]) and frequency domain (mainly PSD, which is analyzed in different frequency bands) features are extracted. Additionally, several nonlinear features are extracted, encompassing Shannon entropy, composite multiscale sample entropy [34], dispersion entropy [35], Katz fractal dimension [36], Kolmogorov complexity [37], and the Hurst index [38], among others. Following the extraction phase, feature selection is undertaken using the mRMR feature selection algorithm [39]. The segments are labeled, designating interfered EEG signals with a 0 and ocular artifact-contaminated EEG signals with a 1. A support vector machine classifier is constructed using these labeled data, which incorporates the computed features and labeling information. For the analysis of new, unseen EEG data, the pre-trained classifier comes into play. It identifies segments contaminated with ocular artifacts, marking them as the positive class (+1), while segments with undisturbed EEG signals are classified as the negative class (0).

### 2.2. Variational Modal Decomposition (VMD)

#### 2.2.1. Fundamentals of Variational Modal Decomposition

By constructing a constrained variational problem and optimally solving it, the VMD technique decomposes the initial signal into numerous modal components, each with distinct bandwidths and frequency centers, facilitating easier signal identification and separation [40]. This approach not only achieves a smoother subsequence encapsulating a variety of frequency scales by mitigating the complexity and nonlinearity of a highly non-smooth time series, but it also addresses the issues of endpoint effects and modal component overlap encountered in the EMD method [41]. The solution procedure of this constrained variational problem is delineated as follows [42]:

(1) Construct the variational problem. Assume that the original signal, *f*, is broken down into *k* components, each having a central frequency within a finite bandwidth. The objective is to minimize the combined estimated bandwidths of all modes. Meanwhile, the condition to meet is that the cumulative sum of all modes should match the original signal.

The corresponding expression is

(1) $$\min_{\left\{u_{k}\},\{w_{k}\right\}}\left\{{\sum_{K}}^{2}\left\|\delta_{t}[(\delta (t)+\frac{j}{\pi t})*u_{k}(t)]e^{-jw_{k}t}\right\|\right\}$$

(2) $$s.t.\sum_{k}u_{k}=f$$

where *δ*(*t*) represents the Dirichlet function. The set $\left\{u_{k}\right\}$ enumerates each component involved in the process, while $\left\{w_{k}\right\}$ denotes the collection of respective central frequencies. *f* signifies the EEG signal that has been contaminated with ocular artifacts. 

(2) Solve the variational problem. The variational problem transforms an unconstrained variational problem, facilitated by utilizing a Lagrange multiplier coupled with a quadratic penalty term. The expression is as follows:

(3) $$\begin{array}{cc} L(\{u_{k}\},\{w_{k}\},\lambda ) & =\alpha \sum_{k}{\left\|\delta_{t}[(\delta (t)+\frac{j}{\pi t})\times u_{k}(t)]e^{-jw_{k}t}\right\|}_{2}^{2}+{\left\|f(t)-\sum_{k}u_{k}(t)\right\|}_{2}^{2}\\ & +<\lambda (t),f(t)-\sum_{k}u_{k}(t)> \end{array}$$

where α represents the quadratic penalty factor, while λ(t) stands for the Lagrange multiplier, the function f(t) corresponds to the EEG signal tainted by ocular artifacts, and < > denotes the inner product operation.

The saddle point of Equation (3), which corresponds to the minimum value in Equation (1), is determined by iteratively updating the IMF components, central frequency, and Lagrange operator using the alternating method of multiplicative operators.

#### 2.2.2. Optimization of VMD Parameters Based on Genetic Algorithm

The VMD decomposition theory [27] posits that before employing VMD for signal decomposition, one must predefine both the number of modal components, *k*, and the quadratic penalty factor, *α*. Existing research [43] has indicated that variations in the settings of *k* and *α* can significantly influence the outcomes of VMD decomposition. Typically, the empirical pre-setting of these two parameters serves as the basis when performing VMD decomposition of signals. However, this approach may not be effective given the complexity and variability inherent in measured signals, potentially hindering the achievement of optimal decomposition results. Choosing appropriate values for *k* and *α* is critical in achieving precise VMD signal decomposition. Given that these two parameters influence each other, optimizing one while holding the other constant can easily result in a local optimization scenario, preventing the acquisition of the optimal decomposition parameter. Therefore, it is imperative to consider a method that optimally adjusts both parameters simultaneously to avoid suboptimal outcomes.

The genetic algorithm (GA) [44], inspired by the principles of natural selection and genetic mechanisms in biology, stands as an adaptive global optimization search algorithm. It offers superior generality, heightened search efficiency, and robust global optimization capabilities compared to other optimization algorithms, such as the ant colony and particle swarm [45]. Central to GA represents a problem solution as a binary coded “chromosome”. Before the algorithm’s execution, a set of “chromosomes” is hypothesized as initial solutions, forming the initial population. This population is progressively optimized through selection, crossover, and mutation processes, gravitating towards a state harboring the optimal solution. Considering the principles of VMD decomposition, it is evident that the modal number *k* and the penalty factor *α*, among its input parameters, have a notable impact on the decomposition results [46]. Due to the GA’s ability to conduct global optimization in the space where the objective function resides, as well as its capacity to address multiple parameters concurrently, this study leverages the GA to optimize both *k* and *α*.

In optimizing VMD parameters using the GA, a sequence of six pivotal steps is necessitated—coding, population initialization, fitness evaluation, selection, crossover, and mutation—culminating in the evolution of a more adeptly adapted population. Central to this optimization process is the third step, “fitness evaluation,” which mandates the establishment of a fitness function. This function acts as the linchpin in steering the computational evolutionary progression, embodying the evaluative metric of the evolutionary computations. The subsequent genetic operations undertaken by all individuals are contingent upon their respective fitness values. In this paper, we construct the fitness function based on approximate entropy (ApEn), which is defined in Section 2.4.

**Figure 2:**
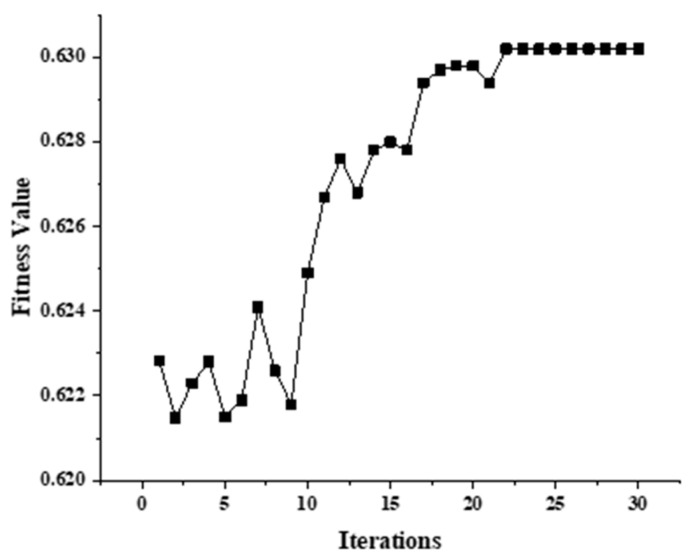
Adaptation–number of iterations variation plot.

This study employs the genetic algorithm (GA) to optimize the variational mode decomposition (VMD) parameters. The parameter configurations are as follows: the modal number *k* ranges from 2 to 10; the penalty factor α varies between 100 and 5000, it iterates 30 times, and the population size is 10. Figure 2 illustrates that the fitness value around the 22nd iteration yields the optimal parameter combination of (*k*, *α*) = (120, 4). Thus, the algorithm identifies (*k*, *α*) = (120, 4) as the optimal parameter set through this search process.

### 2.3. Second-Order Blind Identification Algorithm (SOBI)

The SOBI algorithm [47], also known as second-order blind identification, achieves blind source separation through the joint approximate diagonalization of covariance matrices. Notably, its effectiveness is not contingent upon whether the source signal adheres to a Gaussian distribution, thus offering a versatile solution for various data types. This attribute renders it particularly adept at handling EEG signals, as it can robustly separate signal components while retaining crucial information, promoting more accurate analyses in neural engineering studies.

Assume that the *m*-channel acquired signal is

(4) $$X(t)={\left[x_{1}(t),x_{2}(t),x_{3}(t),\ldots ,x_{m}(t)\right]}^{T}$$

where the original *n*-channel signal is

(5) $$S(t)={\left[s_{1}(t),s_{2}(t),s_{3}(t),\ldots ,s_{m}(t)\right]}^{T}(m\geq n)$$

The mixing matrix is $A_{mxn}$, the instantaneous mixing model is

(6) X(t)=AS(t)

For the model mentioned above, the fundamental procedures of the SOBI blind source separation include the following:

1. Perform whitening on the original data to obtain the whitened data $S\left(t\right)$ and whitened matrix Q. The covariance matrix of W(t) is a unit matrix:
  (7) W(t)=QX(t)
2. Calculate the sampling covariance matrix of W(t) with a fixed delay $\rho \;\epsilon \;\{\rho_{i}|i=1,2,3,\ldots ,n\}$:
  (8) $$D(\rho )=E[W(t+\rho )W^{T}(t)]=AD_{W}(\rho )A^{T}$$
3. The joint approximate diagonalization of each $D\left(\rho \right)$ is performed to compute the orthogonal matrix (V), satisfying
  (9) $$V^{T}D(\rho_{i})V=U_{i}$$
  where {U} is a set of diagonal arrays.
4. Estimate the mixing matrix (A) and the original signal matrix, S(t):
  (10) $$A=U^{-1}Q$$
  (11) S(t)=CX(t)
  where C is the separation matrix, the inverse matrix of A. The source signals associated with the artifacts in $S\left(t\right)$ are processed to obtain a new source signal matrix, $S_{j}(t)$, which in turn reconstructs the signal $X_{j}\left(t\right)=AS_{j}(t)$.

### 2.4. Entropy-Based Identification of Ocular Artifacts

Applying the SOBI algorithm, the components deduced from the results can be conceptualized as distinct signal sources. The process of EEG signal reconstruction involves nullifying components not pertinent to EEG signals, followed by a subsequent reconstruction facilitated by the inverse SOBI transform. Within the domain of information theory, entropy serves as a potent metric in assessing the system’s complexity and the data’s regularity. EEG signals encapsulate the bioelectrical activity occurring intra- and extracellularly within the brain, offering insights into the cerebral state. Contrarily, ocular artifacts originate from activities such as blinking or eye movements, exhibiting a higher complexity than EEG signals. To accurately differentiate between EEG and ocular components, this study leverages approximate entropy, as referenced in [48], as a critical tool in categorization.

The pseudo code of the approximate entropy is presented in Algorithm 1.
**Algorithm 1.** Approximate Entropy (ApEn) CalculationInput:    *S* = [*s*(1), s(2), …, *s*(*N*)] // Time series data of length N    *m*      // Embedding dimension   *r*    // Similarity threshold, typically a fraction of the standard deviation of *S* Output:   *ApEn*  // Calculated Approximate Entropy value Begin: 1. Compute the standard deviation (*SD*) of the time series *S.* 2. Set the similarity threshold *r* = 0.15 ∗ *SD.* 3. Initialize the array of similarity counts *C* to zeros, of length *N* – *m* + *1*. 4. For each embedding dimension *m*′ in {*m*, *m* + *1*}:    a. Construct m′-dimensional vectors *X*(*i*), *i* = 1 to *N* − *m*′ + 1, where each *X*(*i*) = [*s*(*i*), …, *s*(*i* + *m*′ − 1)].    b. For each vector *X*(*i*), *i* = 1 to *N* − *m*′ + 1:       i. Compute the distance *d*[*X*(*i*), *X*(*j*)] for all vectors *X*(*j*), *j* = 1 to *N* − *m*′ + 1, where        *d*[*X*(*i*), *X*(*j*)] = *max*(|*s*(*i* + *k* − 1) − *s*(*j* + *k* − 1)|) for *k* = 1 to *m*′.       ii. If *d*[*X*(*i*), *X*(*j*)] < *r*, increment *C*(*i*) by 1.    c. Compute the logarithmic frequency of similar vector pairs for *X*(*i*) as:     *Phi_m*′(*r*) = (*1*/(*N* − *m*′ + 1)) ∗ *sum*(*ln*(*C*(*i*)/(*N* − *m*′ + 1))) over *i* = 1 to *N* − *m*′ + 1. 5. Calculate the Approximate Entropy *ApEn* as the difference between the logarithmic frequencies of the two consecutive embedding dimensions:    *ApEn* = *Phi_m*(*r*) − *Phi_m* + 1(*r*). End

**Figure 3:**
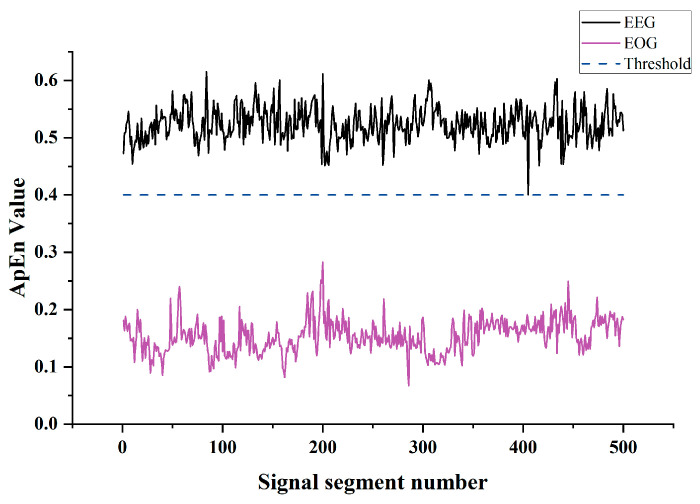
Approximate entropy distribution curve of pure EEG signal vs. EOG signal.

Furthermore, given that experimental data influence the distribution of approximate entropy values, the chosen threshold setting size holds a direct and significant bearing on the efficacy of the artifact removal process. Figure 3 shows the randomly selected 500 segments of pure EEG and EOG signals’ approximate entropy distribution curves. The figure indicates that the approximate entropy of the EEG signals consistently exceeds 0.4. Consequently, the threshold will be established at 0.4 for subsequent artifact processing, which aligns with the parameter settings referenced in the literature [15].

### 2.5. Materials

#### 2.5.1. Simulated EEG Dataset

This study’s simulated dataset [49] includes 4514 clean EEG segments, 2400 ocular artifact segments, and 5598 muscle artifact segments. We derived the simulated data by combining clean EEG signals with ocular artifact signals, as illustrated in the subsequent equation, which represents the following mixing model:

(12) $$X_{mix}=X_{pure\_EEG}+\theta *X_{EOG}$$

where $X_{mix}$ denotes the simulated data obtained after mixing, $X_{pure\_EEG}$ is the pure EEG signal, $X_{EOG}$ signifies the ocular artifact signal, and θ is the signal’s weight. By varying θ, one can modify the signal-to-noise ratio (SNR) of the simulated data, as illustrated in the upcoming equation:

(13) $$SNR=\frac{RMS(X_{pure\_EEG})}{RMS(\theta *X_{EOG})}$$

where RMS denotes the root mean square, defined as shown in the following equation:

(14) $$RMS(X)=\sqrt{\frac{1}{T}XX^{T}}$$

where *T* denotes the number of data points and *X* is the signal.

#### 2.5.2. Real EEG Dataset

The real dataset was obtained from the ISRUC-Sleep public dataset from the Sleep Medicine Center University Hospital of Coimbra [50]. Fifteen subjects were chosen from the sleep apnea syndrome dataset. For every individual, a segment of 3.5 h of sleep data was extracted from their comprehensive sleep records. Each subject’s recording consisted of signals from 19 channels, and 6 EEG channels of the 19 channels were used in this study (F3-A2, C3-A2, O1-A2, F4-A1, C4-A1, and O2-A1), with a sampling frequency of 200 Hz. Experienced sleep specialists labeled the EEG data according to the AASM rules for each 30 s long segment. The data were segmented according to 30 s/segment, and the total number of segments was 6300. Noise within the EEG signal significantly impacts staging accuracy. Therefore, we employed the method proposed in this study to eliminate artifacts from the sleep EEG data.

## 3. Performance Metrics

We conducted a quantitative analysis of the data before and after EOG artifact removal. The primary criteria for evaluation were the extent of effective EEG information retention and the efficacy of ocular artifact elimination [51]. According to the difference in the data, this study adopts different evaluation indexes for the simulated dataset and the real data.

### 3.1. Evaluation of the Simulated EEG Dataset

In this study, we quantitatively analyze the simulated data and the corresponding pure EEG signals after removing the ocular artifacts and evaluate the effectiveness of the ocular artifact removal algorithm in this way. Our evaluation criteria encompass two key aspects: proficiency in effectively eliminating ocular artifacts and the capacity to minimize the distortion of EEG signals. Our research assesses the efficacy of various artifact removal methods using four key metrics: relative root-mean-square error, correlation coefficient, root-mean-square error, and peak signal-to-noise ratio.

(1) Correlation coefficient (*CC*). The correlation coefficient (*CC*) serves as a pivotal indicator to gauge signal distortion in the time domain within our study. It characterizes the degree of correlation between two variables and is particularly effective in quantifying the extent of EEG signal loss. A higher *CC* value indicates a more comprehensive retention of EEG signal information. The following equation defines the *CC*:

(15) $$CC=\frac{Cov(X_{pure\_EEG,}X_{clean})}{\sqrt{Var(X_{pure\_EEG})\cdot Var(X_{pure\_EEG})}}$$

In Equation (18), $X_{pure\_EEG}$ is the pure *EEG* signal and $X_{clean}$ is the simulated data after removing the ocular artifacts.

(2) Relative root-mean-squared error (*RRMSE*). The smaller the value of *RRMSE*, the closer the simulation data after removing the ocular artifact is to the pure EEG signal that constructs the simulation data, and the more completely the ocular artifacts are removed. The following equation defines the RRMSE:

(16) $$RRMSE=\frac{RMS(X_{pure\_EEG}-X_{clean})}{RMS(X_{pure\_EEG})}$$

(3) Mean-square error (*MSE*): MSE evaluates the divergence between the pure EEG signal and the post-ocular artifact removal signal. A smaller *MSE* suggests that after ocular artifact elimination, the signal more closely resembles the pure EEG signal, indicating a superior denoising result.

(17) $$MSE=\frac{1}{N}\sum_{n=1}^{N}{\left(X_{pure\_EEG}(n)-X_{clean}(n)\right)}^{2}$$

(4) Peak signal-to-noise ratio (*PSNR*): The *PSNR* is an evaluation metric usually used to assess signal reconstruction quality in a signal. A higher *PSNR* value indicates less distortion, meaning that the signal closely aligns with the pure EEG data, which signifies a more effective denoising result.

(18) $$PSNR=10\mathrm{lg}\frac{{\left(2^{m}-1\right)}^{2}}{MSE}$$

When evaluating the performance of EEG artifact removal, the value of m is typically set to 8 [52].

### 3.2. Real Data Evaluation Methodology

(1) Given that pure EEG signals cannot be extracted from real data, it is not feasible to quantitatively assess the ocular artifact removal effect using the *RRMSE* and *CC*. Therefore, the power variation (∆*P*) in different frequency bands was used as an index. The effective removal of ocular artifacts can be visualized by comparing the signal waveforms before and after the removal of ocular artifacts, and the distortion of EEG signals is compared by calculating the power spectral density distortion in different frequency bands of the EEG signals after the removal of ocular artifacts (∆*P*), as shown in the following equation:

(19) $$\Delta P=\left|P_{in}-P_{out}\right|$$

where $P_{in}$ represents the power spectral density of the EEG signal before ocular artifact removal and $P_{out}$ signifies the power spectral density of the EEG signal post-ocular artifact elimination.

(2) In addition, since sleep staging is a classification problem, in this study, sleep EEG is categorized into five stages (R, N1, N2, N3, W), which are evaluated by using the overall accuracy (Acc), MF1 (Macro-F1), and WF1 (Weightd-F1), along with the precision (P), recall (R), and F1 score (F1), as defined by the calculation formulas shown below:

(20) $$precision=\frac{TP}{TP+FP}$$

(21) $$recall=\frac{TP}{TP+FN}$$

(22) $$F1=\frac{2*precision*recall}{precision+recall}$$

where *TP* represents the count of positive examples accurately classified, *TN* denotes the count of correctly identified negative examples, *FP* indicates the count of positive examples misclassified, and *FN* tallies the number of negative examples misidentified.

## 4. Results 

### 4.1. Experimental Results of Simulated EEG Data

In this study, 500 data segments correspond to each SNR value in the semi-simulated data. For each segment of data, the ocular artifact removal process was performed using EEMD-ICA [53], SSA-SOBI [23], CWT-KMEANS-SSA [54], VME-DWT [55], and the proposed SVM-IVMD-SOBI method. Each algorithm was run several times on different data for every SNR value, and the effect of the algorithms on ocular artifact removal for different SNR data was evaluated by calculating the mean and standard deviation of the four metrics, namely, RRMSE, CC, MSE, and PSNR, for these data.

**Figure 4:**
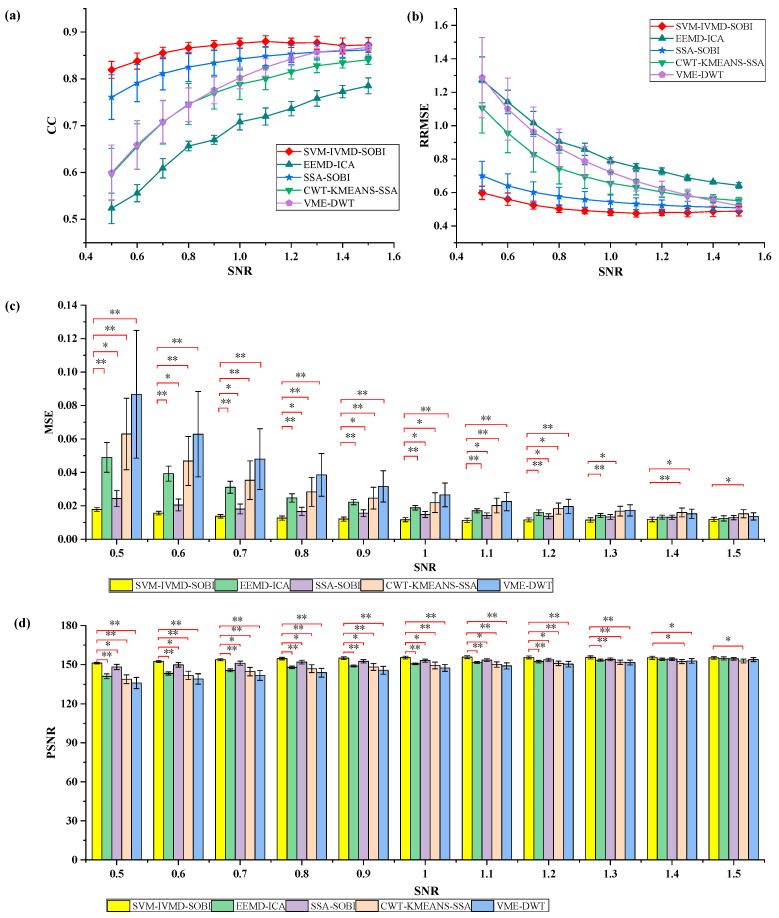
(**a**) Comparison of RRMSE results of five algorithms, (**b**) Comparison of CC results of five algorithms, (**c**) Comparison of MSE results of five algorithms, (**d**) Comparison of PSNR results of five algorithms (*: *p* < 0.05, **: *p* < 0.01).

Figure 4 shows the comparison of the RRMSE, CC, MSE, and PSNR results of EEMD-ICA, SSA-SOBI, CWT-KMEANS-SSA, VME-DWT, and the SVM-IVMD-SOBI method proposed in this study for experiments performed on simulated data. A higher CC value indicates that the EEG signal more closely resembles the clean EEG signal once ocular artifacts are removed. From Figure 4a, it can be seen that with the increase in the SNR value, the CC of the five algorithms corresponds to a gradual increase; when evaluating the five algorithms, the EEMD-ICA algorithm demonstrates a notably lower CC compared to the other four techniques; the SVM-IVMD-SOBI method proposed in this paper is the most stable, and the effect of removing the artifacts is better than that of the other four comparative methods under the conditions of different SNR values, i.e., the signals after removing the electrooculographic artifacts by the approach proposed in this study has the highest degree of similarity to the clean EEG data used to construct the simulated signal, and has the smallest degree of distortion. The smaller the RRMSE value, the cleaner the removal of the ocular artifacts is; based on Figure 4b, as the SNR rises, there is a discernible reduction in the RRMSE values for all five algorithms; in the condition of different SNR values, the RRMSE for the SVM-IVMD-SOBI method put forward in this study is lower than that of the other four methods. The MSE and PSNR results in Figure 4c,d also show that the proposed method in this paper achieves better results on simulation data with different SNR values. Furthermore, to discern any notable differences in the artifact removal efficacy between this study’s proposed method and the four other methods, the results are statistically analyzed using a *t*-test (*p* < 0.05) (shown in Figure 4c,d). The results indicate a notable enhancement in artifact removal using the SVM-IVMD-SOBI method proposed in this study when juxtaposed with the other four methods.

**Figure 5:**
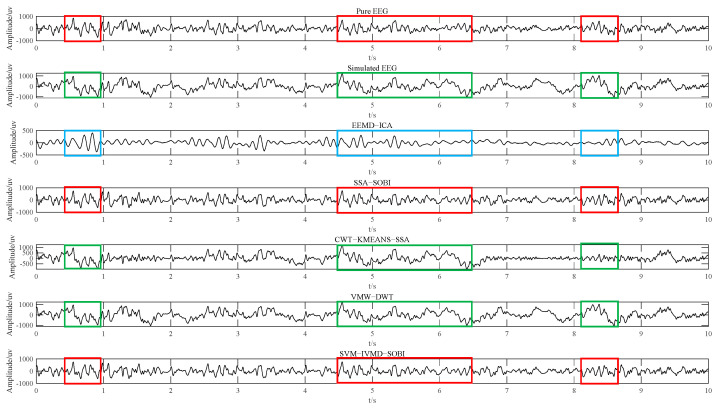
Comparison of ocular artifact removal from simulated data (red markers indicate that EEG signals after artifact removal resemble pure EEG signals; green markers indicate that EEG signals after artifact removal resemble contaminated EEG signals; blue markers indicate that EEG signals after artifact removal are not similar to either pure EEG or contaminated EEG signals).

Figure 5 illustrates the efficacy of the method presented in this study, alongside comparison algorithms, for ocular artifact elimination from simulated data. These data are derived by integrating pure EEG signals with ocular signals at an SNR of 1.0. Figure 5 illustrates that EEG signals processed by the SSA-SOBI and SVM-IVMD-SOBI methods demonstrate similarity to pure EEG signals. Conversely, signals treated by the CWT_KMEANS_SSA and VME_DWT methods show resemblance to contaminated EEG signals. Furthermore, EEG signals processed by the EEMD-ICA method do not exhibit similarity to either clean or contaminated EEG signals. This comparative analysis indicates that compared with the EEMD-ICA, CWT-KMEANS-SSA, and VME-DWT algorithms, the other two algorithms are more effective at removing EEG artifacts, and EEMD-ICA is the worst at removing artifacts. When considering both the CC and RRMSE (${CC}_{Our\;method}>{CC}_{SSA\_SOBI}$ and ${RRMSE}_{Our\;method}<{RRMSE}_{SSA\_SOBI}$), it is evident that this study’s method can remove ocular artifacts in the simulated EEG data more effectively, and the simulated EEG signals after removal are more similar to the pure EEG signals used to construct the simulated data. In other words, our presented approach more efficiently preserves essential signal information, introducing minimal distortion.

### 4.2. Experiment Results of Real EEG Data

**Figure 6:**
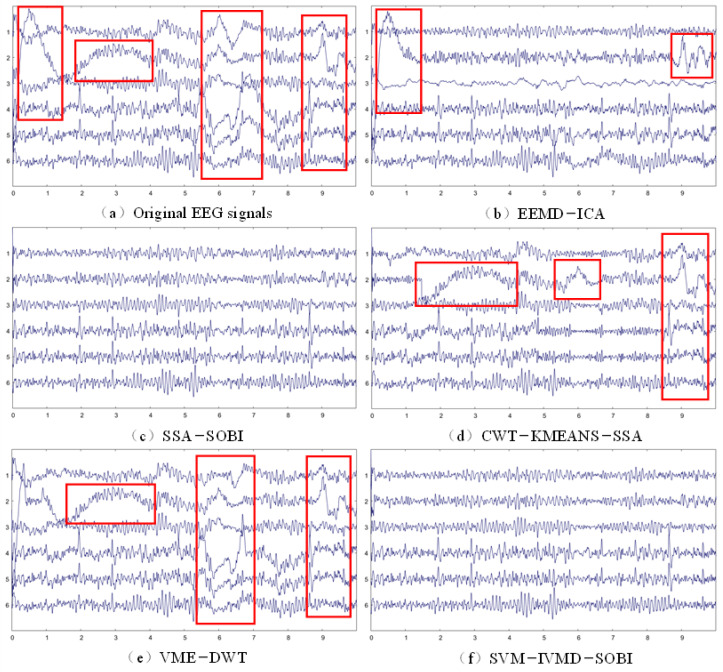
Comparison of the artifact removal effect of different methods in the real dataset (taking the 10 s data of subject one as an example, where 1,2,3,4,5,6 represent channels F3-A2, C3-A2, O2-A2, F4-A1, C4-A1, and O1-A1, respectively; red markers indicate that the presence of artifacts in the original EEG signal and the EEG signals processed by different methods).

In real data experiments, the change in power spectral density before and after artifact removal (∆PSD) offers a quantitative assessment of how well EEG components are retained. In addition, due to the high amplitude and low-frequency characteristics of the EOG artifact itself, the EOG artifact removal effect can also be qualitatively analyzed by observing the waveform graph. Figure 6 demonstrates the waveform graphs before and after processing by different methods. From Figure 6a, it can be seen that the collected raw data produce severe malformed changes in many places due to the influence of EOG artifacts. Figure 6b–f display the processing outcomes of the four comparison algorithms alongside the SVM-IVMD-SOBI method proposed in this study, respectively, from which it is evident that the artifact removal effect of VME-DWT and CWT-KMEANS-SSA is poor, especially the signal after VME-DWT processing, where the artifacts are not removed; SSA-SOBI and the proposed method have a relatively good processing effect.

Previous studies [41,56] assumed that a lower ∆PSD represents a lower level of distortion in the EEG signal after artifact removal. Yet, based on the outcomes from this study’s proposed method, a higher ∆PSD value in the lower frequency band indicates a more effective removal of EOG artifacts from EOG-contaminated EEG signals. Visual comparisons in the graphics further support this observation. Furthermore, examining the ∆PSD results for each channel presented in Table 1, the effectiveness of the SVM-IVMD-SOBI method proposed in this study becomes evident. Specifically, it excels in EOG artifact elimination when contrasted with the PSD variations across frequency bands observed in the SSA-SOBI algorithms.

In a final step to corroborate the efficacy of the method introduced in this study, we applied our proposed methods and methods from EEMD-ICA, SSA-SOBI, and other research to remove ocular artifacts from the ISRUC-Sleep dataset. Subsequently, sleep staging was conducted using the EEG signals post-artifact removal. From the data presented in Table 2, it is clear that compared with the sleep staging results of EEG signals processed by traditional filtering and ICA, removing ocular artifacts can effectively enhance the precision of sleep staging. From the data in Table 2, the method proposed in this study exhibits superior performance. Notably, the ACC, MF1, and WF1 metrics show marked improvement over several other methods. In contrast, the sleep staging outcomes of EEG data treated by EEMD-ICA are the least favorable, falling short compared to results from EEG signals processed solely through filtering and ICA.

## 5. Discussion

As the demand for EEG data analysis grows, enhancing the quality of EEG signal preprocessing has become a focal point of increasing interest [57]. Therefore, a large number of studies have reported how to accurately and efficiently remove ocular artifacts from neural signals. Still, among the many artifact removal methods, most have achieved remarkable results in multi-channel EEG signal processing. How to accurately identify and remove ocular artifacts and maximally preserve neural information in single-channel EEG signal-based processing is still a problem worthy of research and exploration. Therefore, in this paper, we have proposed an automatic recognition and removal algorithm of ocular artifacts combining an SVM, GAVMD, and BSS, and the conducted simulated and real EEG data experiments verify the effectiveness of the proposed method.

In our study, we conducted simulated experiments by formulating signals under various SNR conditions, with the results graphically illustrated in Figure 4. The results indicate that the method presented excels in the efficiency of artifact removal. Specifically, it minimizes the deviation between the reconstructed and clean signals. As substantiated by the upward trend of CC and PSNR alongside the decreasing patterns of MSE and RRMSE with escalating SNRs, it underscores the robustness of our approach. Furthermore, Figure 5 also visualizes the effect of different methods for artifact removal. The distortion level of the reconstructed signal hinges on the successful separation of the ocular and electroencephalographic components. Still, contemporary approaches often indiscriminately process all components during denoising, which can lead to information loss. To address these prevalent issues, we proposed a new method that enhances source separation by using the strategy of dual recognition and dual decomposition. As seen from Figure 5, the denoising effect of the method proposed in this paper is the best under the same conditions due to the effective separation of the ocular and electroencephalographic components.

In our experiments with real data, we utilized six EEG channels. Upon completing artifact removal experiments on single-channel EEG using different methods, we proceeded to perform sleep staging based on the EEG signals after artifact removal. It was observed that while the removal of artifacts does indeed enhance the accuracy of sleep staging, the efficacy of sleep staging predicated on single-channel EEG data remains suboptimal. We speculate that this may be due to the following reasons: single-channel signals offer restricted information and possess a relatively low spatial resolution compared to multichannel signals, potentially insufficient for the intricate requirements of sleep staging. Therefore, we applied our proposed method and comparative algorithms sequentially to EEG data from six channels. Following this, sleep staging was conducted using EEG signals processed through these varied methods. The outcomes of the sleep staging, as delineated in Table 2, provide additional validation of our method’s efficacy in ocular artifact elimination.

It was discerned that EOG signals are chiefly found in the low-frequency band, demonstrating significant low-frequency activity. Consequently, when EEG signals intertwine with ocular artifacts, there is a perceptible alteration in its low-frequency oscillatory information due to ocular oscillations, as shown in Figure 6a. Effective removal of these artifacts from the EEG signals significantly alters the delta and theta frequency bands, with a lesser impact on the alpha and beta bands, a finding echoed in related studies [14,58]. A comprehensive analysis of Figure 6 and Table 1 confirms the rationality of the observed modifications in the power spectral density across different frequency bands post-artifact removal. To further substantiate the efficacy of the GAVMD-SOBI algorithm, it was compared with four other algorithms: EEMD-ICA, SSA-SOBI, CWT-KMEANS-SSA, and VME-DWT. Figure 6b presents the signal reconstructed after artifact removal by the EEMD-ICA method. The figure shows that the artifact removal effect on Channel 2 is insignificant, and the signal distortion on Channel 3 is relatively large. The EEMD-ICA algorithm uses EEMD to decompose the single-channel signal into several IMFs, which satisfies traditional ICA’s input and output conditions. However, due to the modal aliasing phenomenon in the EEMD algorithm and the alteration in signal characteristics instigated by artifact signals, signal reconstruction is prone to errors, hence the relatively poorer artifact removal performance. Further examining Figure 6d,e, it is clear that the CWT-KMEANS-SSA and VME-DWT algorithms fare even worse in removing ocular artifacts, failing to achieve the desired artifact removal outcome. Although facilitating frequency decomposition at varying scales in the frequency domain, the wavelet transformation may lack sufficient resolution for artifacts within a specific frequency range. This inadequacy potentially causes artifact overlapping with the authentic signal frequency, posing a significant challenge in effective discrimination [59]. Moreover, the necessity of boundary expansion during the wavelet transformation to mitigate boundary effects can introduce extra artifacts or compromise the signal’s real part. Compared with the three algorithms EEMD-ICA, CWT-KMEANS-SSA, and VME-DWT, the SSA-SOBI algorithm artifact removal is relatively good. However, SSA is based on the linear combination of signals and matrix decomposition, which may not match the nonlinearity and complexity of actual EEG signals, affecting the effect of artifact removal. Additionally, employing the SSA algorithm necessitates the selection of parameters like window length and overlap rate, where varying values significantly influence the artifact removal effectiveness, coupled with its high computational complexity, requiring extensive computation time.

## 6. Conclusions

In this study, we have proposed an algorithm based on SVM-IVMD-SOBI to eliminate ocular artifacts from single-channel EEG signals. This method combines the robustness of the support vector machine, a refined variant of the variational mode decomposition algorithm bolstered by the genetic algorithm, and second-order blind identification. This combination efficiently removes single-channel ocular artifacts while preserving the maximum possible EEG information. Our empirical analyses underscore the performance of the proposed method. On simulated data, our algorithm demonstrated better performance, evidenced by a smaller root-mean-square error (RRMSE) and a larger correlation coefficient (CC) when compared with four current algorithms: EEMD-ICA, SSA-SOBI, CWT-KMEANS-SSA, and VME-DWT. Furthermore, when applied to real datasets, the algorithm exhibited reduced distortions, particularly in the alpha and beta bands, outperforming the comparison algorithms. Moreover, in the experiment of OSA sleep staging recognition, the proposed method in this paper performs better, achieving a higher overall recognition accuracy and mean F1 score (MF1), thereby attesting to its effectiveness and stability.

While our method yielded favorable outcomes, the SSA-SOBI method’s efficacy was also apparent in our experiments. We believe that further in-depth comparative analysis with the SSA-SOBI method is necessary and therefore consider it as a direction for future research. We are committed to gathering more pertinent data and undertaking detailed evaluations of the various approaches in future studies to provide a more exhaustive comparison.

## Figures and Tables

**Table 1:** Variation in power spectral density (∆P) in different frequency bands after removal of ocular artifacts.

Method		F3	C3	O1	F4	C3	O2	Average
EEMD_ICA [53]	${∆PSD}_{\delta }$	1.3362	1.0019	0.6521	1.3298	1.0479	1.3362	1.0113 ± 0.2948
	${∆PSD}_{\theta }$	0.006	0.0206	0.0236	0.0134	0.0150	0.006	0.0165 ± 0.0064
	${∆PSD}_{\alpha }$	0.0109	0.0121	0.0190	0.0117	0.0132	0.0109	0.0137 ± 0.0030
	${∆PSD}_{\beta }$	0.0068	0.0105	0.0050	0.0093	0.0097	0.0068	0.0076 ± 0.0025
SSA_SOBI [23]	${∆PSD}_{\delta }$	2.5259	1.7836	1.0070	2.3040	1.7182	1.0555	1.7324 ± 0.6235
	${∆PSD}_{\theta }$	0.0174	0.0206	0.0233	0.0220	0.0165	0.0247	0.0207 ± 0.0033
	${∆PSD}_{\alpha }$	0.0033	0.0043	0.0076	0.0038	0.003	0.0082	0.0050 ± 0.0023
	${∆PSD}_{\beta }$	0.0032	0.0053	0.0095	0.0019	0.007	0.009	0.0060 ± 0.0031
CWT_KMEANS_SSA [54]	${∆PSD}_{\delta }$	1.7120	1.2567	0.6645	1.6524	1.2114	0.6845	1.1969 ± 0.4522
	${∆PSD}_{\theta }$	0.1651	0.1575	0.1270	0.1674	0.1451	0.1208	**0.1472 ± 0.0197**
	${∆PSD}_{\alpha }$	0.0320	0.0325	0.0254	0.0341	0.0322	0.028	0.0307 ± 0.0033
	${∆PSD}_{\beta }$	0.0088	0.0050	0.0023	0.0107	0.0076	0.0029	0.0062 ± 0.0034
VME_DWT [55]	${∆PSD}_{\delta }$	0.4991	0.3232	0.1868	0.4589	0.2884	0.1714	0.3213 ± 0.1358
	${∆PSD}_{\theta }$	0.0091	0.0181	0.0172	0.0124	0.0069	0.0177	0.0136 ± 0.0048
	${∆PSD}_{\alpha }$	0.0042	0.0028	0.0017	0.0042	0.0054	0.0067	0.0042 ± 0.0018
	${∆PSD}_{\beta }$	0.0062	0.0046	0.0015	0.0051	0.0029	0.0017	0.0037 ± 0.0019
SVM-IVMD-SOBI	${∆PSD}_{\delta }$	2.5457	1.9539	1.0891	2.5131	1.8873	1.1672	**1.8594 ± 0.6293**
	${∆PSD}_{\theta }$	0.0199	0.0291	0.0333	0.0345	0.0181	0.0376	0.0288 ± 0.0081
	${∆PSD}_{\alpha }$	0.0036	0.0045	0.0037	0.004	0.0055	0.0033	**0.0041 ± 0.0008**
	${∆PSD}_{\beta }$	0.0028	0.0031	0.0054	0.0025	0.0015	0.0061	**0.0036 ± 0.0018**

The bold values in the table show the best results obtained by the proposed method in comparison with other methods.

**Table 2:** Comparison of sleep EEG staging effects after ocular artifact processing using different methods.

	Evaluation Index	W	N1	N2	N3	REM
Filtering, ICA processing	Precision	0.89	0.60	0.75	0.87	0.84
	Recall	0.92	0.52	0.78	0.92	0.75
	F1	0.91	0.56	0.77	0.90	0.79
	ACC: 0.804, MF1: 0.784, WF1: 0.802					
EEMD-ICA [53]	Precision	0.85	0.54	0.68	0.86	0.73
	Recall	0.90	0.33	0.77	0.89	0.68
	F1	0.87	0.41	0.72	0.87	0.71
	ACC: 0.763, MF1: 0.716, WF1: 0.754					
SSA-SOBI [23]	Precision	0.90	0.64	0.72	0.86	0.94
	Recall	0.97	0.49	0.75	0.91	0.84
	F1	0.93	0.55	0.74	0.88	0.89
	ACC: 0.820, MF1: 0.798, WF1: 0.816					
CWT-KMEANS-SSA [54]	Precision	0.86	0.62	0.74	0.90	0.85
	Recall	0.91	0.43	0.79	0.95	0.81
	F1	0.88	0.51	0.76	0. 92	0.83
	ACC: 0.815, MF1: 0.781, WF1: 0.809					
VME-DWT [55]	Precision	0.85	0.67	0.76	0.91	0.77
	Recall	0.87	0.45	0.86	0.93	0.78
	F1	0.86	0.54	0.81	0. 92	0.78
	ACC: 0.809, MF1: 0.780, WF1: 0.803					
SVM-IVMD-SOBI	Precision	0.87	0.68	0.81	0.92	0.91
	Recall	0.94	0.53	0.85	0.97	0.81
	F1	0.90	0.60	0.83	0.94	0.85
	ACC: 0.854, MF1: 0.824, WF1: 0.850					

## Data Availability

A publicly available simulated dataset was used in this work [39] and a real dataset was used in this work [40].
